# Lesion- and Patient-Related Variables May Provide Additional Clues during Dermoscopic Assessment of Blue Nevi—A Retrospective Cohort Study

**DOI:** 10.3390/cancers14081920

**Published:** 2022-04-10

**Authors:** Martyna Sławińska, Grażyna Kamińska-Winciorek, Urszula Balicka, Anton Żawrocki, Roman J. Nowicki, Michał Sobjanek, Enzo Errichetti

**Affiliations:** 1 Department of Dermatology, Venereology and Allergology, Faculty of Medicine, Medical University of Gdańsk, 80-214 Gdańsk, Poland; rnowicki@gumed.edu.pl (R.J.N.); msobjanek@gumed.edu.pl (M.S.); 2 Department of Bone Marrow Transplantation and Oncohematology, Skin Cancer and Melanoma Team, Maria Sklodowska-Curie National Research Institute of Oncology, Gliwice Branch, 44-102 Gliwice, Poland; dermatolog.pl@gmail.com; 3 Dermatological Students Scientific Association, Department of Dermatology, Venereology and Allergology, Faculty of Medicine, Medical University of Gdańsk, 80-214 Gdańsk, Poland; urszulamariabalicka@gmail.com; 4 Department of Pathology, Ceynowa Hospital, 84-200 Wejherowo, Poland; anton.zawrocki@gumed.edu.pl; 5 Institute of Dermatology, “Santa Maria della Misericordia” University Hospital, 33100 Udine, Italy; enzoerri@yahoo.it

**Keywords:** dermoscopy, dermatoscopy, videodermoscopy, blue nevus, nevus, melanoma

## Abstract

**Simple Summary:**

Blue nevi (BN) are dermal dendritic melanocytic proliferations which may be congenital or acquired. Due to wide clinical and dermoscopic presentation, their diagnosis may sometimes be difficult, especially if the history of lesion occurrence is unknown. Little is known about the correlation between lesion- and patient-related variables and dermoscopic features of blue nevi. The aim of the study was to analyze dermoscopic features of blue nevi, with particular regard to structures whose prevalence has not been previously reported, and to investigate the possible influence of selected clinical variables on dermoscopic presentation. Our findings provide new insights into the dermoscopic structures observed in blue nevi and their variability according to patient’s phototype and lesion size/localization.

**Abstract:**

**Background:** Little is known about the correlation between lesion- and patient-related variables and the dermoscopic features of blue nevi. The aim of the study was dermoscopic analysis of blue nevi in association with patient- and lesion-related variables, with a special interest in structures whose prevalence has not been previously reported. **Methods:** This was a double-center, retrospective study, which included the analysis of histopathologically confirmed blue nevi (*n* = 93). **Results:** There was no difference in the frequency of the observed dermoscopic features according to patients’ gender and age. Pink structureless areas were more common in patients with I/II Fitzpatrick skin phototypes as well as in the patients with photodamaged skin, while blue prominent skin markings over brownish/blue-gray background occurred exclusively in patients with phototype III. Structures of previously unreported prevalence in blue nevi were skin-colored circles (present in 32.3%), gray circles (2.2%), follicular ostia with no pigmentation (18.4%; present exclusively on the face), blue skin markings over brownish background (present in 18.2%; detected only on the limbs) and dark brown polygons (one lesion located on the lower extremity). **Conclusion:** Dermoscopic presentation of blue nevi may vary according to the patient’s phototype and lesion size/localization rather than gender and age.

## 1. Introduction

Blue nevi (BN) are dermal dendritic melanocytic proliferations which may be congenital or acquired. Due to the wide clinical and dermoscopic presentation, their diagnosis may sometimes be difficult, especially if the history of lesion occurrence is unknown. Although BN are not particularly rare, few studies concerning their dermoscopic features have been published [1,2,3]. Additionally, the literature data is even more limited when it comes to the association of specific dermoscopic features of BN and clinical variables, with only one study investigating the possible influence of age and anatomical localization of the lesions [2]. On the other hand, there is a lack of information about the variability of dermoscopic features of BN according to the age and size of the lesion, the localization of photodamaged skin, and the patient’s skin phototype. The aim of the study was to analyze clinical and dermoscopic features of BN, with particular regard to structures whose prevalence has not been previously reported, as well as to evaluate the possible influence of selected clinical variables of dermoscopic presentation.

## 2. Methods

This retrospective observational descriptive study based on medical documentation included consecutive patients who underwent blue nevi excision between 1 June 2016 and 1 October 2020 in two dermatology centers. Clinical and dermoscopic pictures were captured using FotoFinder videodermoscope (non-polarized dermoscopy; video camera Medicam 800HD) with ultrasound gel as an immersion fluid (×20 magnification). All BN were measured on a dermoscopic image with the use of the FotoFinder measuring tool. Exclusion criteria were a lack of clinical/videodermoscopic images or histopathological result, low quality images, larger congenital plaque-type BN (diameter over 25 mm), as well as agminated BN with overlapping features of adjacent lesions, and collision lesions. Clinical and dermoscopic pictures were evaluated by two certified dermoscopists (MS and GKW) according to the structures described in the last International Dermoscopy Society (IDS) consensus by Kittler et al. [4]. Based on close-up images, morphology of the lesions was categorized as flat, partially/slightly elevated, or nodular. In case of discrepancy, the final score for particular cases and structures was obtained based on the decision of the third evaluator (MSo). Additionally, previously non-reported dermoscopic features have been introduced in the analysis based on personal observations. Clinical data was obtained based on medical records. Data analysis was carried out using statistical software (Statistica 13.1, Statsoft.). κ statistic and percentage of positive concordance were calculated for interobserver agreement analysis; *p* < 0.05 was considered statistically significant.

## 3. Results

### 3.1. Clinical Features

**Figure 1:**
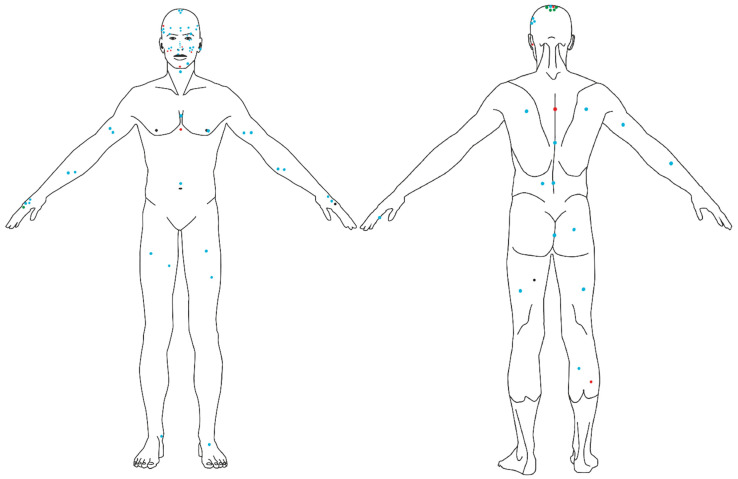
Anatomical distribution of different histopathological subtypes of blue nevi analyzed in the study. Blue dots represent common blue nevi; red dots, combined blue nevi; green dots, cellular blue nevi; black dots, desmoplastic blue nevi.

The analysis included 93 histopathologically confirmed BN from 83 patients (54 female/29 male, age range: 5–93; median age–46; mean age–45.5), including 19 special subtypes (12 combined nevi dermal and blue, 5 cellular BN, and 2 desmoplastic BN). The patients were of Polish origin and I-III Fitzpatrick skin phototype (I–2 [2.4%]; II–45 [54.2%]; III–33 [39.8%]; in three cases phototype data was missing). In 17 (18.3%) lesions, the history was unknown. For 9 (9.7%) lesions, the lesion was present for less than 12 months; in 21 cases (22.6%) the lesion occurred between 1 and 5 years before the procedure; in 8 (8.6%), 5–10 before; in 38 cases (40.9%), more than 10 years before excision. Of the lesions, 41 (44.1%) were flat, 26 (28.0%) were partially/slightly elevated, and 26 (28.0%) were nodular. The mean size of the lesions was 4.3 mm (range 0.5–30.1 mm) and the median was 3.8 mm. Of the lesions, 42 (45.2%) were located on the skin with signs of ultraviolet photodamage. BN were most commonly located on the face (*n* = 31; 33.3%), followed by the scalp (*n* = 18; 19.6%), upper extremity (*n* = 11; 11.8%), lower extremity (*n* = 11, 11,83%), and acral areas, i.e., dorsal surface of the hands and feet (*n* = 9; 9.7%), back (*n* = 7; 7.5%), chest (3; 3.23%), neck (1; 1.1%), abdomen (1; 1.1%), and anogenital area (1.1%). Figure 1 shows the anatomical distribution of the lesions in the studied group.

### 3.2. Dermoscopic Features

The most common dermoscopic patterns observed were structureless blue (*n* = 60; 64.5%) followed by structureless gray (*n* = 44; 47.31%) and structureless dark brown (*n* = 38; 40.9%).

There was no difference in the frequency of the observed dermoscopic features according to patients’ gender and age. Pink structureless areas occurred in 40.4% of patients with lower (I/II) skin phototype compared to 15.1% of patients with III skin phototype (*p =* 0.025), while blue prominent skin markings over brownish/blue-gray background occurred exclusively in patients with phototype III (*p =* 0.061).

When comparing the presence of analyzed dermoscopic structures according to lesion localization, there was a significant correlation for gray clods (which occurred in 12.2% of lesions located on the face/scalp and in 36.4% of lesions located on the limbs, but have not been observed in other anatomical regions; *p* = 0.007) and white clods (present in 36.4% of lesions on the trunk and 9.1% on the limb, but not observed on the face/scalp; *p* = 0.0006). Follicular ostia with no pigment occurred exclusively on the face/scalp (present in 18.4% of lesions; *p* = 0.030), while blue prominent skin markings over brownish/blue-gray background were detected only on the limbs (18.2%; *p =* 0.004). Thin reticular lines were observed more commonly on the limbs (13.6%) compared to the face (2.0%) and not detected in other anatomical regions (*p =* 0.010). Radial peripheral lines were present mostly on the trunk (27.3%) and were less prevalent on the limbs (4.5%), face/scalp (4.1%), and not detected in other anatomical areas (*p =* 0.026).

When analyzing the prevalence of dermoscopic features according to lesion clinical morphology, significant association was found for white structureless areas (present in 53.8% of nodular lesions, 19.2% of partially elevated lesions, and 17.1% of flat lesions; *p =* 0.002), structureless pink (present in 42.3% of nodular lesions, 30.8% of partially elevated lesions, and 12.2% of flat lesions; *p =* 0.018), skin-colored circles (present in 46.2% of nodular lesions, 46.2% of partially elevated lesions, and 14.6% of flat lesions; *p =* 0.005), dotted vessels (present in 53.8% of nodular lesions, 23.1% of partially elevated lesions, and 14.6% of flat lesions; *p =* 0.002), linear vessels (present in 23.1% of nodular lesions, 4.9% of flat lesions, and absent in partially elevated lesions; *p =* 0.006), and polymorphic vessels (present in 23.1% of nodular lesions, 4.9% of flat lesions, and absent in partially elevated lesions; *p =* 0.006).

Pink structureless areas were more common on photodamaged skin (38.1% vs. 15.7%; *p =* 0.026). No significant association was found between the presence of particular dermoscopic structures and lesion age. Structureless blue areas were observed in 81.3% of larger lesions (with a size larger than the median of 3.83 mm) and 46.7% of smaller lesions (*p =* 0.001). Gray structureless areas, white structureless areas, and dotted vessels were more prevalent in smaller lesions (≤3.83 mm), compared to larger ones (>3.83 mm) (62.2% vs. 33.3%, *p =* 0.010; 42.2% vs. 14.6%, *p =* 0.006; 42.2% vs. 14.6%, *p =* 0.006, respectively).

### 3.3. Structures of Previously Unreported Frequency

**Figure 2:**
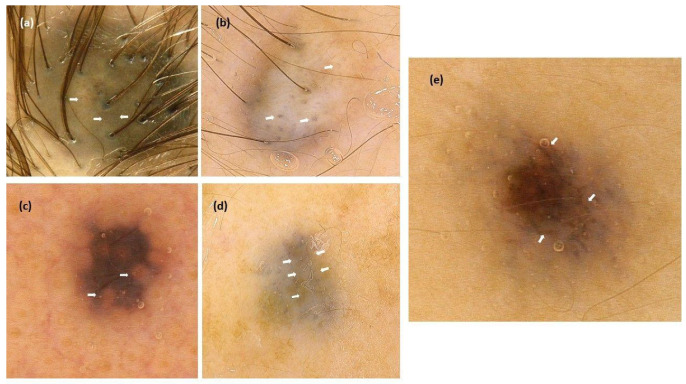
Examples of dermoscopic structures observed in the study, whose prevalence was not previously reported in the context of blue nevi (arrows). (**a**). Skin-colored circles; (**b**). Gray circles; (**c**). Follicular ostia with no pigmentation; (**d**). Blue prominent skin markings over blue-gray background; (**e**). Polygons.

The presence of skin-colored circles was noted in 32.3% of patients (30/93). These structures occurred more commonly in nodular/partially elevated BN compared to flat lesions (present in 46.2% of nodular lesions, 46.2% of partially elevated lesions, and 14.6% of flat lesions; *p =* 0.005). Gray circles were observed in 2.2% (2/93). Follicular ostia with no pigmentation were observed in 18.4% and occurred exclusively on the face. Blue prominent skin markings over brownish//blue-gray background were detected only on the limbs (present in 18.2%). Dark brown polygons were detected in one lesion located on the lower extremity. Previous studies did not report the presence or frequency of the mentioned structures (Figure 2).

### 3.4. Combined Blue Nevi, Cellular Nevi, and Desmoplastic Nevi

In our study, there were 12 combined BN, located mostly on the face (5/12) and scalp (3/12). Main dermoscopic patterns were structureless gray (9/12), structureless light brown (8/12), and structureless blue (6/12). Cellular nevi were located on the scalp/acral areas and dermoscopically, mainly presented with a structureless light brown, structureless gray, and structureless white pattern.

In the studied group, there were two desmoplastic BN located on acral areas and on the lower extremity, which presented with the combination of structureless blue, dark brown, gray, and white areas, as well as the combination of light brown and pink structureless areas, respectively.

Table 1 shows the frequency of dermoscopic features in the study group according to patient-related variables and Table 2 according to lesion-related variables. Table 3 shows a summary of key study findings.

## 4. Discussion

Although structureless bluish to steel-blue pigmentation is considered a prototypical dermoscopic feature of BN, it is not a constant variable. Similarly, the history—previously considered to be of the utmost importance in the management of BN—has been shown to have a lower decisional weight as it is often undetermined, though patients may sometimes report lesion growth or change [5,6,7,8]. In fact, in this study, in more than half of the lesions, the history of their occurrence reported by the patient was below five years or unknown. In such circumstances, broader knowledge on dermoscopic clues associated with particular clinical variables could, at least in some cases, help to make decisions about patient management.

One of the important features considered during the dermoscopic assessment of nevi is the patient’s gender and age, but only one study examined those associations [2,9]. Di Cesare et al. [2] observed no significant correlation between the age of the patients and the presence of local dermoscopic features, in line with our findings.

Sun exposure and skin phototype may affect the dermoscopic presentation of benign and malignant melanocytic skin tumors, yet the association between skin phototype/photodamage signs and dermoscopic features of BN has not been studied in detail so far [10,11,12]. Thus, our study provides new knowledge in this regard.

Prominent skin markings in their classical form present as linear furrows, which are lighter in color than the rest of the lesion [13]. They have been defined as one of the diagnostic clues of melanoma in situ, but also described in Reed nevus, melanocytic nevus, seborrheic keratosis/solar lentigines, Bowen disease, and basal cell carcinoma [14]. Blue prominent skin markings over brownish/blue-gray background, which do not match the classical definition given above, in our observation represent a quite typical, and previously unreported feature of BN. Notably, both classical prominent skin markings and blue prominent skin markings over brownish/blue-gray background turned out to occur mainly on the limbs.

Another variable possibly influencing the dermoscopic pattern of nevi is anatomical localization, yet studies on BN provided diverse findings. In a study by Di Cesare et al. [2]. BN located on the scalp/face region showed significant association with whitish scar-like areas and a multichromatic pattern (*p* = 0.06). Similarly, in our study, white structureless areas were observed most commonly on these anatomical areas, but the association did not reach statistical significance. In contrast, Ferrara et al. [1] reported a higher frequency of white color for lesions located on the limbs.

The management of tiny, pigmented lesions remains a diagnostic challenge, especially if lesion history is short or unknown [15]. In such cases, in vivo confocal microscopy may improve the diagnostic accuracy [16]. Ferrara et al. [1] found no dermoscopy/size association in the analyzed BN. In our study, larger lesions more commonly presented with structureless blue areas, whereas gray structureless areas, white structureless areas, and dotted vessels were more prevalent in smaller lesions.

In a study by Ferrara et al. [1], most lesions were either white-blue (22/52; 42.3%) or blue (19/52; 36.5%). In a study by Di Cesare et al. [2], a monochromatic diffuse blue pigmentation was present in 20% of the analyzed lesions (19/95). Additionally, the dichromatic combination of blue with brown, gray, or black was present in 54.7% of cases, while a blue color occurred in some (unspecified) portion of lesions with multichromatic patterns. In our study, a structureless blue color occurred in 60 lesions (64.5%), in 35 (37.6%) with the dichromatic combination, most commonly as blue and light brown (8; 8.6%), blue and dark brown (7; 7.5%), or blue and gray (6; 6.5%). Multichromatic/polychromatic pigmentation (three colors or more) were detected in from 9.6–15.8% cases in previous studies and 46.2% in our study [1,2]. From a practical point of view, it is important to discuss the prevalence of some features that occur rarely in BN, but they may pose diagnostic difficulties due to their occurrence in melanoma.

One of these structures are linear pigmented projections at the periphery of a melanocytic lesion—linear streaks and pseudopods (bulbous projections). While a symmetrical distribution at the periphery of the whole lesion is usually associated with Spitz/Reed nevus, an asymmetric distribution pattern raises the suspicion of a melanoma [17]. Peripheral radial lines (peripheral streaks) were present in 6.5% (6/93) in our study, compared to 4.2% (4/95) in a study by Di Cesare et al. [2]. We have observed them in flat/partially elevated lesions but not in nodular BN. Additionally, one case revealed the presence of pseudopods. Besides the above-mentioned studies, there are several case reports in the literature describing BN with peripheral streaks [18,19]. According to Sakamoto et al. [18], their presence correlates with the distribution of focally aggregated and isolated spindle cells with melanin in the upper dermis visible in histopathological assessment. Another finding to be considered are pigmented networks, which seem to be a rare observation in BN. Di Cesare et al. [2] reported network-like patterns in 1.1% (1/95). In our study, pigmented networks (lines reticular) occurred in six cases (6.5%) of flat/partially elevated lesions, but not in nodular BN. One of the cases in our series also revealed the presence of polygons (also referred to as rhomboids, zigzag pattern, or angulated lines), which are known to be associated also with lentigo maligna on the face, lentiginous melanoma developing over sun-damaged skin, or nevus-associated melanoma in small- and medium-sized congenital nevi [12,20,21].

Blood vessels in BN were reported by Di Cesare et al. [2] in 12.6% of cases (12/95) and the pattern was most commonly polymorphic. In our study, vascular pattern was detected in 33.7%; vessels were most commonly detected in lesions presenting with a structureless light brown pattern. Polymorphic vessels—defined as the presence of two or more vessel shapes—were observed only in adult patients, predominantly with the I/II skin phototype, and were significantly more common in nodular vs. flat/partly elevated lesions. The higher prevalence of vessels in our study could be, at least in part, explained by the use of a videodermoscope instead of a hand-held dermoscope.

In the spectrum of BN, there are also special subtypes, including combined blue nevi, cellular nevi, and desmoplastic nevi. Combined BN are formed by two populations of melanocytes and dermoscopically often present with a combination of blue/brown colors. In a recent study by IDS, mainly including flat or slightly elevated lesions located on the trunk or head and neck [3], predominant dermoscopic patterns of such a type of BN were structureless blue in combination with brown clods, lines, or skin-colored/brown structureless areas. In comparison to analyzed melanoma instances, the blue part of combined BN was more often well-defined and located in the center of the lesion. In a study by Schweizer et al. [22] (*n* = 36), the majority of combined BN was either located on the trunk (44.4%) or on the face (30.6%). Dermoscopic features present in combined BN, and not observed in analyzed comparators from the melanoma series, were homogeneous intensity of pigmentation and regularly arranged brown globules. In our study, there were 12 combined BN, located mostly on the face and scalp, and main dermoscopic patterns were structureless gray, structureless light brown, and structureless blue.

Interestingly, we have found a relatively high prevalence of circles, including skin-colored circles (present in 32.3%) and gray circles (in 2.2%), not mentioned in previous original studies [1,2]. It is possible that the use of a videodermoscope in our study allowed us to detect them more easily.

Study limitations include the retrospective character of the study, lack of a control group including other possible melanoma mimickers and melanoma, and lack of dermoscopic–histopathological correlation analysis. Some blue lesions presenting with a long, stable history and typical dermoscopic features were not excised, so the frequency of analyzed structures may not fully mirror the whole spectrum observed in daily practice. Images performed with polarized videodermoscopy were not available, so we did not include structures observed in the polarized mode in our study. It would be important to include polarized dermoscopy images in further studies, as some structures (e.g., white shiny lines) can be visualized only by using this setting [23]. Both centers involved in the study are specialized in pigmented skin lesions and melanoma, which may be responsible for selection biases.

## 5. Conclusions

In conclusion, the dermoscopic presentation of BN may vary according to a patient’s phototype and lesion size/localization rather than gender and age. The study provides new insights into the dermoscopic structures observed in blue nevi and their variability according to clinical variables. Future studies would be needed to validate the new criteria against a control group consisting of lesions that mimic blue nevi.

## Figures and Tables

**Table 1:** The frequency of dermoscopic features in the study group according to the patient-related variables.

Dermoscopic Structure	Gender		Age at Excision			Skin Phototype						
	Female *n* = 56	Male *n* = 37	*p*	>18 Years *n* = 85	≤18 Years *n* = 8	*p*	I/II *n* = 47	III *n* = 33	*p*	*n*	*kappa*	Concordance between Both Readers: Positive Agreement, %/Presence According to Both Readers, %
Structureless blue	37	23	0.869	56	4	0.609	28	30	**0.025**	60	1	
Structureless light brown	22	15	0.924	35	2	0.606	20	14	0.949	37	1	
Structureless dark brown	26	12	0.259	35	3	0.862	20	17	0.703	38	0.9778	98.9/40.9
Structureless gray	25	19	0.673	38	6	0.204	28	15	0.257	44	1	100/47.3
Structureless white	16	10	0.941	26	0	0.152	18	7	0.145	26	1	100/28.0
Structureless black	0	1	0.834	0	1	0.138	1	0	0.874	1	1	100/1.1
Structureless pink	14	10	0.981	23	1	0.633	19	5	**0.025**	24	1	100/25.8
Dots brown	3	0	0.405	2	1	0.613	2	1	0.782	3	1	100/3.2
Clods blue	2	0	0.666	2	0	0.403	1	1	0.618	2	1	100/2.6
Clods gray	11	3	0.22	12	2	0.760	9	4	0.548	13	0.9567	98.9/14.0
Clods white	3	4	0.566	5	2	0.208	3	4	0.664	7	1	100/7.5
Clods brown	6	4	0.734	9	1	0.667	8	2	0.242	10	0.9463	98.9/10.8
Circles gray	1	1	0.666	2	0	0.403	1	1	0.618	2	1	100/2.2
Circles skin-colored	19	11	0.843	27	3	0.949	17	13	0.940	30	0.9499	97.9/30.1
Lines white reticular	1	2	0.713	3	0	0.613	2	1	0.781	3	1	100/3.2
Vessels dotted	11	15	0.050	24	2	0.828	18	8	0.243	26	1	100/28.0
Vessels coiled	2	1	0.815	3	0	0.613	3	0	0.362	3	0.8517	98.9/3.2
Vessels linear	5	3	0.811	8	0	0.804	6	2	0.510	8	1	100/8.6
Vessels curved	3	3	0.922	6	0	0.981	4	2	0.977	6	1	100/6.4
Vessels linear irregular	2	2	0.924	4	0	0.776	4	0	0.218	4	1	100/4.3
Vessels polymorphic	4	4	0.811	8	0	0.804	7	1	0.159	8		
Vessel distribution unspecific	12	11	0.508	22	1	0.682	15	8	0.553	23	1	100/24.7
Vessel distribution clustered	0	0	0	0	0	0	0	0	0	0	0	100/0
Vessel distribution regular	0	1	0.834	1	0	0.138	1	0	0.874	1	1	100/1.1
Polygons–dark brown	1	0	0.834	0	1	0.138	1	0	0.874	1	1	100/1.1
Follicular ostia with no pigment	5	4	0.954	9	0	0.732	6	3	0.831	9	1	100/9.7
Blue skin marking over brownish/blue-gray background	2	2	0.924	4	0	0.776	0	4	0.061	4	1	100/4.3
Lines reticular and thin brown	4	0	0.254	3	1	0.776	2	2	0.845	4	1	100/4.3
Lines reticular thick	2	0	0.666	2	0	0.403	1	1	0.618	2	0.6618	98.9/1.1
Lines radial peripheral	3	3	0.922	5	1	0.981	3	3	0.977	6	1	100/6.5
Pseudopods	0	1	0.834	1	0	0.138	1	0	0.874	1	1	100/1.1

**Table 2:** The frequency of dermoscopic features in the study group according to the lesion-related variables.

Dermoscopic Structures	Anatomical Localization					Lesion Morphology				Lesion on Photodamaged Skin			Lesion History						Lesion Size		
	Scalp/ Face *n* = 49	Trunk *n* = 11	Limb *n* = 22	Other *n* = 11	*p*	Flat *n* = 41	Partially/ Slightly Elevated *n* = 26	Nodular *n* = 26	*p*	No *n* = 51	Yes *n* = 42	*p*	Un-known *n* = 17	<1 Year *n* = 9	1–5 Years *n* = 21	5–10 Years *n* = 8	>10 Years *n* = 38	*p*	≤3.83 mm * n = 48	>3.83 mm * n = 45	*p*
Structureless blue	33	7	13	7	0.927	29	17	14	0.369	32	28	0.861	11	6	14	6	23	0.950	39	21	0.001
Structureless light brown	18	1	12	6	0.057	12	10	15	0.067	21	16	0.929	9	1	6	3	18	0.175	15	22	0.127
Structureless dark brown	17	7	9	5	0.358	13	14	11	0.196	18	20	0.322	7	3	12	3	13	0.517	19	19	0.962
Structureless gray	21	7	11	5	0.649	18	13	13	0.843	23	21	0.793	6	4	10	3	21	0.683	16	28	0.010
Structureless white	16	2	4	4	0.477	7	5	14	**0.002**	12	14	0.414	5	2	5	1	13	0.730	7	19	0.006
Structureless black	0	0	1	0	0.353	0	1	0	0.272	0	1	0.922	0	0	0	0	1	0.833	0	1	0.974
Structureless pink	14	3	6	1	0.607	5	8	11	**0.018**	8	16	**0.026**	7	2	5	2	8	0.618	10	14	0.371
Dots brown	1	0	2	0	0.337	1	1	1	0.930	3	0	0.313	0	0	1	0	2	0.773	1	2	0.955
Clods blue	2	0	0	0	0.607	1	0	1	0.624	1	1	0.563	0	0	1	0	1	0.832	2	0	0.503
Clods gray	6	0	8	0	**0.007**	4	6	4	0.331	6	8	0.493	1	0	4	1	8	0.395	5	9	0.317
Clods white	0	4	2	1	**0.0006**	2	3	2	0.602	6	1	0.190	2	0	4	0	1	0.129	1	6	0.097
Clods brown	7	1	2	0	0.561	4	2	4	0.645	5	5	0.991	2	0	2	2	4	0.587	2	8	0.075
Circles gray	1	0	0	1	0.355	0	0	2	0.072	1	1	0.563	0	0	0	0	2	0.565	0	2	0.447
Circles skin-colored	15	5	6	4	0.736	6	12	12	**0.005**	13	17	0.188	4	2	7	1	16	0.388	15	15	0.994
Lines white reticular	2	1	0	0	0.489	0	1	2	0.217	3	0	0.313	0	0	1	1	1	0.513	0	3	0.218
Vessels dotted	11	5	7	3	0.462	6	6	14	**0.002**	14	12	0.911	5	3	7	1	10	0.836	7	19	0.006
Vessels coiled	2	0	1	0	0.808	0	1	2	0.217	1	2	0.864	1	0	0	1	1	0.458	1	2	0.955
Vessels linear	6	0	0	2	0.157	2	0	6	**0.006**	5	3	0.933	1	0	1	2	4	0.365	2	6	0.228
Vessels curved	5	0	0	1	0.315	2	0	4	0.067	2	4	0.503	1	0	0	2	3	0.149	2	4	0.614
Vessels linear irregular	4	0	0	0	0.289	1	0	3	0.090	1	3	0.476	0	0	1	1	2	0.632	1	3	0.564
Vessels polymorphic	6	0	1	1	0.508	2	0	6	**0.006**	5	3	0.933	1	0	1	2	4	0.365	2	6	0.228
Vessel distribution unspecific	10	4	6	3	0.704	6	7	10	0.084	12	11	0.957	5	2	5	3	8	0.875	5	18	0.002
Vessel distribution regular	0	0	0	1	0.057	0	0	1	0.272	1	0	0.922	0	0	1	0	0	0.483	0	1	0.974
Polygons—dark brown	0	0	1	0	0.353	1	0	0	0.527	1	0	0.922	0	0	1	0	0	0.483	1	0	0.974
Follicular ostia with no pigmentation	9	0	0	0	**0.030**	6	2	1	0.320	4	5	0.759	0	0	4	1	4	0.287	7	2	0.193
Blue skin markings over brownish//blue-gray background	0	0	4	0	**0.004**	2	1	1	0.971	3	1	0.753	1	0	0	0	3	0.557	1	3	0.564
Lines reticular and thin brown	1	0	3	0	**0.010**	2	2	0	0.381	1	3	0.476	2	0	2	0	0	0.187	3	1	0.656
Lines reticular thick	1	0	1	0	0.780	1	1	0	0.624	1	1	0.563	1	0	0	0	1	0.735	2	0	0.503
Lines radial peripheral	2	3	1	0	**0.026**	4	2	0	0.272	3	3	0.859	1	1	1	1	2	0.909	3	3	0.733
Pseudopods	0	1	0	0	0.057	0	0	1	0.272	1	0	0.922	0	1	0	0	0	0.051	1	0	0.974

* Median size from all lesion measurements.

**Table 3:** Key observations from the study.

There was no difference in frequency of the observed dermoscopic features according to patients’ gender and age. Pink structureless areas in BN occurred more commonly in patients with a lower (I/II) skin phototype as well as in lesions found on the skin with the signs of photodamage. Blue prominent skin markings over brownish background occurred exclusively in phototype III. No significant association was found between the presence of particular dermoscopic structures and lesion age. Gray clods occurred significantly more commonly in BN located on the limbs while white clods in BN found on the trunk. Follicular ostia with no pigment occurred only in BN on the face/scalp. Thin reticular lines were observed more commonly on the limbs compared to the face and not detected in other anatomical regions. Lines radial peripheral were present mostly on the trunk. White structureless areas, pink structureless areas, and skin-colored circles were observed mainly in nodular BN. Among the features, which prevalence was not previously reported in the context of BN, the most common findings in this study were skin-colored circles (32.3%), follicular ostia with no pigment (18.4%), and blue prominent skin markings over brownish/blue-gray background (18.2%). Polymorphic vessels considering nonspecific signs of malignancy may also be encountered in benign BN, especially in nodular lesions in patients with I/II skin phototype.

## Data Availability

All data used in the study are available from the corresponding author upon request.
